# Automated Inferior Vena Cava Filter Retrieval Requests and Hematology Liaison Improves Retrieval and Reduces the Use of Temporary Inferior Vena Cava Filters

**DOI:** 10.1055/s-0040-1713176

**Published:** 2020-06-13

**Authors:** Lara N. Roberts, Thoraya Ammar, Julia Czuprynska, Roopen Arya, C. Jason Wilkins

**Affiliations:** 1 Department of Haematological Medicine, King's Thrombosis Centre, King's College Hospital National Health Service Foundation Trust, London, United Kingdom; 2 Department of Radiology, King's College Hospital NHS Foundation Trust, London, United Kingdom

The use of inferior vena cava (IVC) filters has expanded despite the limited evidence demonstrating their benefit for any indication. 1 2 Retrievable filters are now widely utilized to minimize complications associated with permanent siting. 2 However, IVC filters are often left in situ. 3 4 National guidelines of United Kingdom (published in 2012) for the management of venous thromboembolism (VTE) therefore recommended early planning and documentation of IVC filter retrieval, with regular review of the planned approach. 4 A subsequent audit of 2012 practice revealed 52 filters were placed at King's College Hospital, with 14 (27%) subsequently retrieved. Of the remaining patients, there were nine (17%) subsequent deaths, one documented decision for the filter to remain in situ and 32 (62%) patients lost to follow-up. To address this, we planned a single-center multidisciplinary quality improvement project involving interventional radiology and hematology aimed at improving the IVC filter retrieval rate, implemented in February 2015. Internal communications were utilized to raise staff awareness. The interventions included are as follows:

- An updated IVC filter policy incorporating prespecified indications for IVC filter placement, an emphasis on the importance of early filter retrieval when no longer required and radiology nursing preassessment of patients prior to retrieval.
- Automated electronic IVC filter retrieval requests (generated with the IVC filter insertion request at the time of submission on the electronic patient records (EPR; Allscripts Sunrise, Chicago, Illinois, United States) as a placeholder within Computerized Radiology Information System (CRIS) with concomitant automated electronic notification to the hematologist (via e-mail) generated by the EPR at the time of IVC filter request.
- Monthly radiology reports of all IVC filter insertions and retrievals sent to hematology (as a back up to the automated notification system) and to facilitate tracking patient outcomes as per below.
- Hematological review of individual patient electronic records post-IVC filter insertion to document retrieval recommendations, track patient outcomes, and arrange face-to-face review when appropriate.
- Letters to the local general practitioner (GP) for “out-of-area” patients discharged with an IVC filter in situ, providing advice on IVC filter retrieval and recommending local referral when appropriate.

This transitioned the responsibility for IVC filter retrieval decision making from the requestor to hematology/radiology. Additionally, we noted an increase in request volume for IVC filters and increased hematological review of electronic patient records prior to IVC filter insertion (when feasible) to combat this from 2017.

We report the impact of the initial interventions (1–5) for all IVC filters ( *n* = 263) inserted from January 2015 to February 2018, and the number of IVC filters avoided from 2017 to 2019. The majority of patients were male ( *n* = 157, 59.7%), with a mean age of 59 (±18) years. The indication for filter placement and source of IVC filter request are summarized in the Table 1 . Over this time period, Celect (Cook Medical, Indiana, United States) and Denali (Bard, Arizona, United States) IVC filters were in use, with open ended retrieval time windows.

**Table 1:** Summary of indications for IVC filter placement and referral source

Indication, *n* (%)	
Acute VTE with contraindication to anticoagulation	133 (50.5)
Primary VTE prevention in trauma	62 (23.6)
Perioperative thromboprophylaxis	32 (12.2)
Secondary VTE prevention a	22 (8.3)
Primary VTE prevention in neurosurgical patients	5 (1.9)
Recurrent VTE on therapeutic anticoagulation	5 (1.9)
Massive PE	4 (1.6)
Referral source, *n* (%)	
Trauma	87 (33.0)
Neurosurgery	81 (30.7)
Internal medicine	33 (12.5)
Hepatobiliary surgery	22 (8.3)
General surgery	20 (7.6)
Stroke medicine	15 (5.7)
Other	5 (1.9)

Abbreviations: IVC, inferior vena cava; PE, pulmonary embolism; VTE, venous thromboembolism.

a Patients with recurrent VTE on long-term anticoagulation admitted to hospital with active bleeding.

Of note, the most frequent indication for IVC filter placement was acute VTE with a contraindication to anticoagulation in 50.5%. All trauma patients with an IVC filter inserted for primary VTE prevention were unable to receive prophylactic anticoagulation due to active bleeding/high rebleeding risk associated with their injuries. Sixty-six (25%) patients were discharged to an out-of-area residence with an IVC filter in situ. Letters were written to the GP for all out-of area patients but the filter outcome remains unknown for 53 (20.1%) patients; these were excluded from further analysis. Of the remainder ( *n* = 210), 37 (17.6%) patients had a documented decision for the IVC filter to remain in situ, and there were 37 (17.6%) unrelated deaths within 6 months of insertion (of which 5 had a documented decision for IVC filter to remain in situ). IVC filter retrieval was attempted for 137 (65.2%) patients and successful in 87.4% ( *n* = 119, including two with >1 attempt). On table venography was performed immediately prior to attempted filter retrieval in all cases. Filter retrieval occurred at a median of 85 days (interquartile range [IQR]: 43–150) postinsertion. Two (0.8%) local patients were lost to follow-up with the IVC filter in situ, despite multiple attempts at review. Seven (5.1%) patients had IVC filter thrombus seen at cavogram (with retrieval deferred) and a further 10 patients had a failed first attempt at retrieval (5.0% of insertions and 7.4% of attempted retrievals). Of these 17 patients, a subsequent decision to leave the filter in situ was made with 10 patients, two filters were later successfully retrieved, 2 patients had further unsuccessful retrieval attempts, and 2 patients were lost to further follow-up with one unrelated death.

The described interventions led to a significant increase in IVC filter retrievals compared with 2012 (odds ratio [OR] = 2.7, 95% confidence interval [CI]: 1.4–5.4, *p* = 0.003) with a significant reduction in loss to follow-up (OR = 0.07, 95% CI: 0.03–0.16, *p* < 0.001). An additional benefit was increased formal documentation of decisions for IVC filters to remain in situ (17.6% compared with 1.9% in 2012; *p* < 0.001).

A key limitation is the lack of outcome data for the patients discharged out of area with an IVC filter in situ; this represented a significant proportion of our cohort due to the regional provision of trauma/neurosurgical services at King's College Hospital. Unfortunately, despite writing to the local GP with recommendations regarding IVC filter retrieval and a request to inform us of outcome, this occurred for a minority of patients (19.6%). The exclusion of these patients from further analysis may have erroneously inflated the retrieval rate. Reanalysis including this group and assuming none of these IVC filters were retrieved reduces the retrieval rate to 62.3% (119/190); this remains significantly improved compared with 2012. Further improvement in communication across the regional network is required. Our approach is comparable with other quality improvement programs in the United States with retrieval rates improving to 58–75%. 5 6 7 8

Despite the limited evidence for IVC filters, there is increasing use particularly for VTE prevention. 1 We noted an upward trend in the number of filters placed each year ( Fig. 1 ). From 2017, hematology played an informal but active role in ensuring appropriate usage of IVC filter placement with the impact on IVC filter insertions shown in the Fig. 1 . In 2019, the IVC filter policy was updated to mandate hematological approval in the absence of proximal deep vein thrombosis (within 4 weeks) with a contraindication to anticoagulation, or major trauma patients with pelvic fractures and a continuing contraindication to thromboprophylaxis beyond 72 hours. This led to a reduction in total number of filters placed for the first time since the quality improvement program launched, with a significant increase in the number of requests declined compared with 2018 ( *p* = 0.02). We note the recent negative findings of the randomized controlled trial of IVC filters for primary VTE prevention following major trauma. 9 Restricting IVC filters for primary VTE prevention is therefore justified, and revising the IVC filter policy to acknowledge this will further reduce the use of IVC filters. This will require further engagement with key requestors and agreement of an alternate strategy, such as use of intermittent pneumatic compression devices and regular review of bleeding risk, to enable early initiation of anticoagulant thromboprophylaxis. Nevertheless, our multidisciplinary approach with IVC filter tracking led to significantly improved IVC filter retrieval rates, improved documentation of decision-making, and latterly reduced IVC filter insertions.

**Fig. 1:**
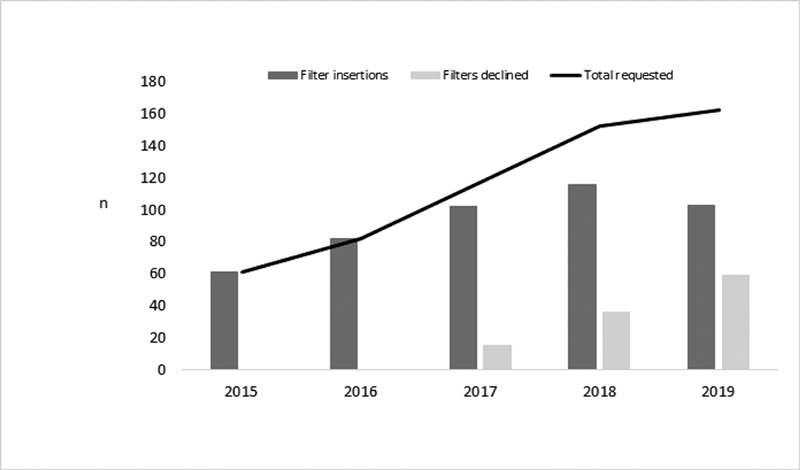
Number of IVC filters requested, inserted and declined 2015 to 2019. IVC, inferior vena cava.

## References

[1] Weinberg I Appropriate use of inferior vena cava filtersAvailable at:https://www.acc.org/latest-%ADin-%ADcardiology/articles/2016/10/31/09/28/appropriate-%ADuse-%ADof-%ADinferior-%ADvena-%ADcava-%ADfilters. Accessed June 24, 2019

[2] Geerts W Selby R Inferior vena cava filter use and patient safety: legacy or science? Hematology (Am Soc Hematol Educ Program) 2017 2017 01 686 692 29222322 10.1182/asheducation-2017.1.686 PMC6142573

[3] Angel L F Tapson V Galgon R E Restrepo M I Kaufman J Systematic review of the use of retrievable inferior vena cava filters J Vasc Interv Radiol 2011 22 11 1522 1.53E6 22024114 10.1016/j.jvir.2011.08.024

[4] NICE.Venous thromboembolic diseases: diagnosis, management and thrombophilia testing 2012. Available at:https://www.nice.org.uk/guidance/cg144/evidence/full-guideline-pdf-186726352. Accessed June 24, 2019

[5] Lynch F C A method for following patients with retrievable inferior vena cava filters: results and lessons learned from the first 1,100 patients J Vasc Interv Radiol 2011 22 11 1507 1512 21903414 10.1016/j.jvir.2011.07.019

[6] Minocha J Idakoji I Riaz A Improving inferior vena cava filter retrieval rates: impact of a dedicated inferior vena cava filter clinic J Vasc Interv Radiol 2010 21 12 1847 1851 21035356 10.1016/j.jvir.2010.09.003

[7] Rogers F B Shackford S R Miller J A Wu D Rogers A Gambler A Improved recovery of prophylactic inferior vena cava filters in trauma patients: the results of a dedicated filter registry and critical pathway for filter removal J Trauma Acute Care Surg 2012 72 02 381 384 22327980 10.1097/TA.0b013e3182447811

[8] Winters J P Morris C S Holmes C E A multidisciplinary quality improvement program increases the inferior vena cava filter retrieval rate Vasc Med 2017 22 01 51 56 27811236 10.1177/1358863X16676658

[9] Ho K M Rao S Honeybul S A multicentre trial of vena caval filters in severely injured patients N Engl J Med 2019 381 04 328 337 31259488 10.1056/NEJMoa1806515

